# Bipedality in chacma baboons (*Papio ursinus griseipes*) in Gorongosa National Park: a multi-contextual, socially-relevant and demographically-structured behavior

**DOI:** 10.1007/s10329-026-01277-w

**Published:** 2026-07-17

**Authors:** K. Chappell-Smith, D. I. Cavallaro, S. Carvalho

**Affiliations:** 1 Institute of Human Sciences, University of Oxford, Oxford, UK; 2 School of Anthropology, Pontificia Universidad Católica de Chile, Santiago de Chile, Chile; 3 Department of Science, Gorongosa National Park, Sofala, Mozambique; 4 PALEO Research Group, CIBIO-BIOPOLIS, Campus de Vairão, Vairão, Portugal

**Keywords:** Human evolution, Bipedalism, Sociality, Age-sex structures, Primate models

## Abstract

Obligate bipedalism is a defining trait of the human lineage, yet the selective pressures underlying its emergence and fixation remain debated, particularly within the woodland-grassland mosaic environments associated with early hominins. Modern semi-terrestrial primates inhabiting comparable ecosystems provide a useful model for evaluating candidate functional contexts of early bipedal behavior in the human lineage. Here, we present the first systematic description of bipedal behavior in chacma baboons (*Papio ursinus griseipes*). Using all-occurrence sampling across two troops of baboons in Gorongosa National Park, Mozambique, we recorded 231 bipedal bouts over 12 observation days. Bipedalism was predominantly postural and most frequently occurred in vigilance contexts. Several social contexts of bipedalism were recorded, including play, grooming, socio-sexual mounting, and attempted infant snatching. Arboreal bipedalism was rare and primarily linked to foraging. Rates and contexts of bipedalism varied across age-sex classes. Infants and juveniles exhibited higher bipedalism rates than other classes and expressed the broadest bipedal context repertoire. Vigilance was the only context prompting bipedalism across all age-sex classes. We discuss these patterns to evaluate how multi-contextual, socially-relevant and age- and sex-structured bipedalism behavior in baboons may inform reconstructions of early hominin bipedalism.

**Supplementary Information:**

The online version contains supplementary material available at https://doi.org/10.1007/s10329-026-01277-w.

## Introduction

Obligate terrestrial bipedalism distinguishes modern human locomotion from that of other extant African apes (*Pan* and *Gorilla*), which are semi-terrestrial, quadrupedal knuckle-walkers (Darwin 1871; Doran 1993; Fleagle 1984; Pontzer 2017). However, the evolutionary origins of this mode of locomotion have been the subject of debate for decades (Fleagle et al. 2026). A popular hypothesis is that bipedalism emerged and became fixed in the human lineage as an adaptation to the progressive expansion of dry, open grassland environments across the late Miocene to early Pleistocene (Cerling et al. 2011; Dart 1925; Domínguez-Rodrigo 2014; Levin 2015), evolving hand-in-hand with terrestrial locomotion and foraging (Hunt 1994, 1996; Napier 1967; Sockol et al. 2007). However, recent reconstructions of early hominin paleoenvironments place them in mosaic woodland-grassland savanna ecosystems with persistent (albeit temporally and spatially variable) woody cover across the last 6 million years (Bobe and Carvalho 2019; Cerling et al. 2011; Negash et al. 2024). These heterogeneous ecosystems would have presented unique challenges and selective pressures for hominins, and new perspectives suggest bipedalism may have evolved unlinked from terrestriality within these contexts (Drummond-Clarke et al. 2022; Hammond et al. 2025; Richmond et al. 2001; Takemoto 2004). In light of this evidence, it is crucial to reconstruct the selective pressures that may have led to the emergence and fixation of bipedalism in the human lineage within woodland-grassland mosaic environments and in the context of potential hominin semi-terrestriality (Drummond-Clarke et al. 2022; Hammond et al. 2025).

Several hypotheses on why bipedalism was selected for in the human lineage have arisen through the study of modern non-human primates (“primates” hereafter) which occasionally adopt bipedal postures or move on two legs (Carvalho et al. 2012; Druelle and Berillon 2014; Drummond-Clarke et al. 2022; Gebo 1992; Hewes 1961; Hunt 1994; Jolly 1970; Larson 2018; Rose 1976; Wrangham 1980; Youlatos 2002). Key hypotheses for the proposed functions of bipedalism in hominins include alertness/vigilance (Day 1986), foraging (including in arboreal and semi-terrestrial contexts) (Drummond-Clarke et al. 2022; Jolly 1970; Pilbeam and Jacobs 1978; Rose 1976; Wrangham 1980), using tools for defence or hunting (Dart 1959), carrying of food, infants or tools (Amaral 2022; Carvalho et al. 2012; Etkin 1954; Hewes 1961; Isaac 1978; Lovejoy 1981; Washburn 1967), coping with water shortages (Mitchell et al. 2009), and wading or foraging in water (Hardy 1960; Wrangham et al. 2009) (see Fleagle et al. 2026 for a review).

The estimated and generally accepted time of the emergence of hominin habitual bipedalism (sensu Stamos and Alemseged 2023) is ~ 4.2 million years ago, with the appearance of *Australopithecus* in the fossil record (Alemseged 2023; Stamos and Alemseged 2023) (c.f. The ~ 11.6 Ma *Danuvius guggenmosi*, a miocene ape from the region of Bavaria, shows the potential for a degree of habitual bipedalism [Böhme et al. 2019]; however, this remains under debate [Williams et al. 2020]). Many traits that appeared in the human lineage following this (including brain expansion, spoken language, tool use and cooperation) were deeply connected to human sociality, relying not solely on bipedal morphology, but on hominin social bonding and ‘theory of mind’ (Boyd and Richerson 2009; Dunbar 2010a; Hrdy 2011; Tomasello 2020). However, with the exception of a few authors (e.g. Hill et al. 2009; Jablonski and Chaplin 1992), the majority of hypotheses have given little consideration for the role of sociality in the emergence of bipedalism, nor the potential role of the emergence of bipedalism in facilitating the evolution of social traits in the human lineage. In addition, while infant carrying hypotheses position females as the primary drivers of the emergence of bipedalism (Amaral 2022; Etkin 1954; Lovejoy 1981), no hypotheses appear to consider that different age and sex classes could have performed bipedalism for different reasons, therefore more strongly selecting for the evolution of this trait in hominins.

Here, we provide a first description and quantification of the contexts in which bipedalism occurs in chacma baboons (*Papio ursinus*), including an exploration of social contexts and an investigation into the age-sex differences in bipedal behaviors. Although phylogenetically more distant from humans than the African apes, quadrupedal and pronograde in posture (Rose 1976; D’Août et al. 2014), baboons are semi-terrestrial and occupy woodland-savanna mosaic environments similar to those associated with Plio-Pleistocene hominins (Elton 2006; King 2022). Furthermore, certain hominins and papionins often appear together in the Pliocene and early Pleistocene fossil record (e.g., Bobe et al. 2002; Frost et al. 2020), and thus their behavior and ecology may have been shaped by similar environmental pressures (Elton 2006). Despite these parallels, quantitative and qualitative data on baboon bipedal behavior remains scarce (Nagel 1973; Rose 1976). Our study aimed to address this gap. Specifically, we build on the findings of Rose (1976) that among olive baboons (*Papio anubis*), bipedalism occurs in a range of contexts including foraging as well as social contexts such as play and greeting. In addition, Rose (1976) noted that the frequency and contexts of bipedal behaviors differed across age-sex classes, with infants showing a high incidence of bipedal behavior across several contexts compared to other age groups, and bipedalism involving infant carrying occurring mostly in association with adult females (Rose 1976). Our study assessed whether similar patterns are present in chacma baboons and, thus, across species.

## Methods

### Study site and subjects

The study was conducted in Gorongosa National Park (GNP; Fig S1), a 4,067 km^2^ protected area in central Mozambique. The park’s landscape is characterized by woodland-grassland mosaic vegetation and the presence of a major body of water, lake Urema (Daskin et al. 2016). At least five species of primates (Carvalho et al. 2025) currently inhabit the park alongside multiple herbivore and carnivore species (Stalmans et al. 2019, 2022). Today’s diversity in GNP reflects the ongoing conservation efforts of the Gorongosa Restoration Project in response to the collapse of many wildlife populations that took place during and after the Mozambican civil war (1977–1992) (Pringle and Gonçalves 2022; Stalmans et al. 2019). However, at the time of this study (July–August, 2018), GNP still represented a heavily carnivore-depleted ecosystem (Atkins et al. 2019; Bouley et al. 2018; Pringle 2017). For instance, leopards (*Panthera pardus*) –one of baboon’s most frequent predators (Altmann and Altmann 1970; Isbell et al. 2018)– were extirpated during the Mozambican civil war and only reintroduced in 2019 (Gorongosa National Park 2024; Pringle and Gonçalves 2022; Stalmans et al. 2019). Despite the effects of this conflict on other park species, genetic analyses of GNP’s baboons have not yielded evidence of significant population contractions during their recent demographic history (Ferreira da Silva et al. 2025).

Approximately 230 baboon troops (Stalmans et al. 2022), ranging between approximately 30–108 individuals in size, inhabit GNP. Studies of the taxonomy of Gorongosa’s baboons have suggested they are best classified as greyfooted chacma (*Papio ursinus griseipes*) (Martinez et al. 2019; Santander et al. 2022), although they show signals of admixture with yellow baboons (*Papio cynocephalus*) (Ferreira da Silva et al. 2025; Martinez et al. 2019; Santander et al. 2022). Data collection for this study was carried out on two baboon troops: the Chitengo troop and the Woodland troop. The Chitengo troop’s home range encompasses Chitengo Camp, a ~ 1.7 km^2^ fenced area that serves as a base for park staff and researchers. Chitengo has open woodland borders and an internal environment characterized by sparse vegetation, man-made structures and persistent human activity, making it habituated to human presence.

During the 2018 field season, when the data for this study was collected, the Chitengo troop was composed of approximately 40 members. Troop age-sex composition was not recorded during the 2018 field season. However, during the 2019 field season, the Chitengo troop was recorded as composed of 11 adult males, 11 adult females, one subadult/large juvenile female, approximately 16 juveniles, and an indeterminate number of infants (Muschinski 2025). The Woodland troop was composed of approximately 88 individuals during the time of this study (Hammond et al. 2022) and its home range encompasses ~ 7.5 km^2^ of medium-to-dense Vachellia–Combretum woodland (Lewis-Bevan et al. 2025). The composition of the Woodland troop was not recorded at the time of this study, and it was in the process of becoming habituated during the study period (Hammond et al. 2022; Lewis-Bevan et al. 2025). We did not identify individuals in either troop.

### Data collection

We used the application Animal Observer (v.1.2.2; Calliaud 2016) on an Apple iPad (4th generation) for the collection of behavioral data. Data collection took place over 12 days during July and August of 2018 via all-occurrence sampling, a method suited for the collection of rare behaviors (Bateson and Martin 2021). The Woodland and Chitengo troops were studied for four days and eight days, respectively (Table 1). Only one sampling session was conducted per day. Each sampling session corresponded to one observation period on one day for one troop. Each sampling session began when the focal troop was spotted and ended when the focal troop stopped being visible or after 540 min (nine hours) of observation had gone by. Because of the collection methods, the sampling sessions are of different lengths (n = 12; mean: 368.0 min; range: 57.7–540 min). Sampling sessions in the Chitengo troop were, on average, shorter than the Woodland troop sampling sessions. Therefore, although the Chitengo troop was observed for eight days and the Woodland troop was observed for four days, they have comparable observation hours (Chitengo: 39.3 h, Woodland: 34.3 h). Eight of the sampling sessions (four from each troop) began between 6:00 and 8:30 am, and the remaining four sampling sessions (all from Chitengo troop) began between 1:00 and 3:00 pm. Within each sampling session, all instances of bipedal postures and locomotion were recorded. We followed Rose (1976) in recording and including in our analyses bipedal postures performed both with and without a support substrate. However, differentiating between the two in each bipedal event was precluded by visibility in the majority of cases. Therefore, we do not report this information. Baboons were not individually identifiable. If several troop members simultaneously performed bipedal bouts, all of them were recorded.

**Table 1:** Overall and troop-specific sampling effort

Troop	Complete observation time (hours)	Number of sampling sessions	Mean and range of sampling session length (minutes)
Woodland	34.3	4	513.8 (501.1–540.0)
Chitengo	39.3	8	295.1 (57.7–540.0)
Overall	73.6	12	368.0 (57.7–540.0)

Additionally, we recorded:

1. The age-sex category to which the individual performing the bipedal behavior belonged to, specified as follows (based on Altmann et al. 1981): infant/juvenile, subadult male, subadult female, adult male and adult female. Sex was not recorded for infants and juveniles due to challenges in identifying it from a distance. We also recorded the age-sex category of each visible troop member in any given sampling session every 30 min, starting from the beginning of the sampling session.
2. The apparent situation or context in which the bipedal bout occurred. These situations were described and, where the same situations appeared in repeated occasions, their total number was quantified.
3. Whether bipedalism occurred on the ground, arboreally (on tree branches above three meters of height) or on an object slightly elevated from the ground (on tree branches under three meters of height or on another structure under three meters of height).

Multiple bipedal bouts performed by the same individual within five seconds or less of each other in the same context were counted as a single bout.

### Data analysis

To assess whether bipedalism rates differed across age–sex classes, we tested whether each age–sex class showed different numbers of bipedal bouts after accounting for “class-specific exposure”: the amount of time each age-sex class was available for observation per session. This was necessary because the number of visible individuals differed across age–sex classes and sessions, and we were not able to characterize the overall composition of each troop during the study. Therefore, raw counts of bipedal bouts could not be directly compared across age–sex classes without accounting for differences in observation opportunity. To estimate class-specific exposure, we first counted the number of visible individuals in each age-sex class every 30 min of each sampling session. We call those 30 min intervals “scans”. We then estimated the mean number of individuals visible in each age–sex class (*c*), during each session (*i*) for each troop (*t*) as:

1 $$\overline{N}_{ict} = \frac{1}{{K_{i} }}\sum\limits_{k = 1}^{{K_{i} }} {N_{kict} } $$

where $\overline{N}_{ict}$ represents the mean number of visible individuals in age–sex class* c* during session *i* in troop *t*, *K*_*i*_ represents the number of 30 min scans conducted during session *i* and *N*_*kict*_ represents the number of visible individuals in age–sex class *c* during scan *k*, session *i*, troop *t*. Observations of individuals whose age-sex class could not be identified were excluded. Next, we calculated the exact duration of each sampling session as the difference between its end and start timestamps, first in minutes and then converted this value to decimal hours. Therefore, sessions did not need to last a whole number of hours. For example, the shortest session (57.7 min) contributed 0.96 h of observation time. Finally, class-specific exposure for each session (*E*_*ict*_) was estimated in hours by multiplying the mean number of visible individuals in each age–sex class during the session ($\overline{N}_{ict}$) by the exact session duration in decimal hours (*D*_*it*_):

2 $$ E_{ict} = \overline{N}_{ict} \times D_{it} $$

Next, we created a dataset aggregating bipedalism observations at the level of session, troop, and age–sex class. This dataset was analyzed in the model specified below (Eq. 3). Each row of this dataset represented one age–sex class in one sampling session for one troop, and contained the number of bipedal bouts observed (*Y*_*ict*_) and the class-specific exposure (*E*_*ict*_) for that class x session x troop combination. Age–sex classes that had exposure hours over zero during a session but did not perform any bipedal bouts were retained as zero-count observations. Rows with zero exposure were excluded because those age–sex classes were not available for observation during that session. The model dataset comprised 42 session × troop × age–sex class observations.

Bipedal bout counts per session, troop and age-sex class were analysed using a quasi-Poisson generalized linear model with a log link to test whether bipedalism rates differed across age–sex classes while controlling for troop identity and accounting for unequal observation opportunity among age–sex classes. Let *Y*_*ict*_ denote the number of bipedal bouts observed for age–sex class *c* during sampling session *i* in troop *t*, and *E*_*ict*_ the corresponding class-specific exposure in hours, the model was specified as:

3 $$ \begin{gathered} Y_{ict} = quasi{ - }Poisson(\mu_{ict} ,\phi ) \hfill \\ \log (\mu_{ict} ) = \log (E_{ict} ) + \beta_{0} + \beta_{c} + \gamma_{t} \hfill \\ \end{gathered} $$

where $\mu_{ict}$ is the expected number of bipedal bouts, age–sex class ($\beta_{c}$) and troop ($\gamma_{t}$) were included as fixed effects, and the natural logarithm of class-specific exposure was included as an offset. Thus, model estimates should be interpreted as age-sex-class specific bipedalism rates: the expected number of bipedal bouts per hour of class-specific exposure for each age–sex class, adjusted for troop identity. A quasi-Poisson model was selected to account for overdispersion in the count data. All analyses were done in R (v. 4.4.2; R Core Team 2024).

### Ethical note

This work was carried out with ethical clearance issued to the Paleo-Primate Project by Gorongosa National Park (permit number PNG/DSCI/C79/2017). All data collection was observational and conducted in the primates’ natural habitats. Researchers did not come into contact with any animals.

## Results

### Summary

In total, 231 bouts of bipedal behavior were observed across 12 days of observation. Of these, 162 (70.1%) were postural bouts and 69 (29.9%) were bouts of bipedal locomotion. Overall, 212 bouts (91.8%) were on the ground, 12 (5.2%) occurred on raised objects (among them car tops, tents and low tree branches) and 7 bouts (3.0%) were arboreal. Of the seven arboreal bouts, six were observed in the Woodland troop and one was observed in the Chitengo troop.

### Bipedalism by context

**Fig. 1:**
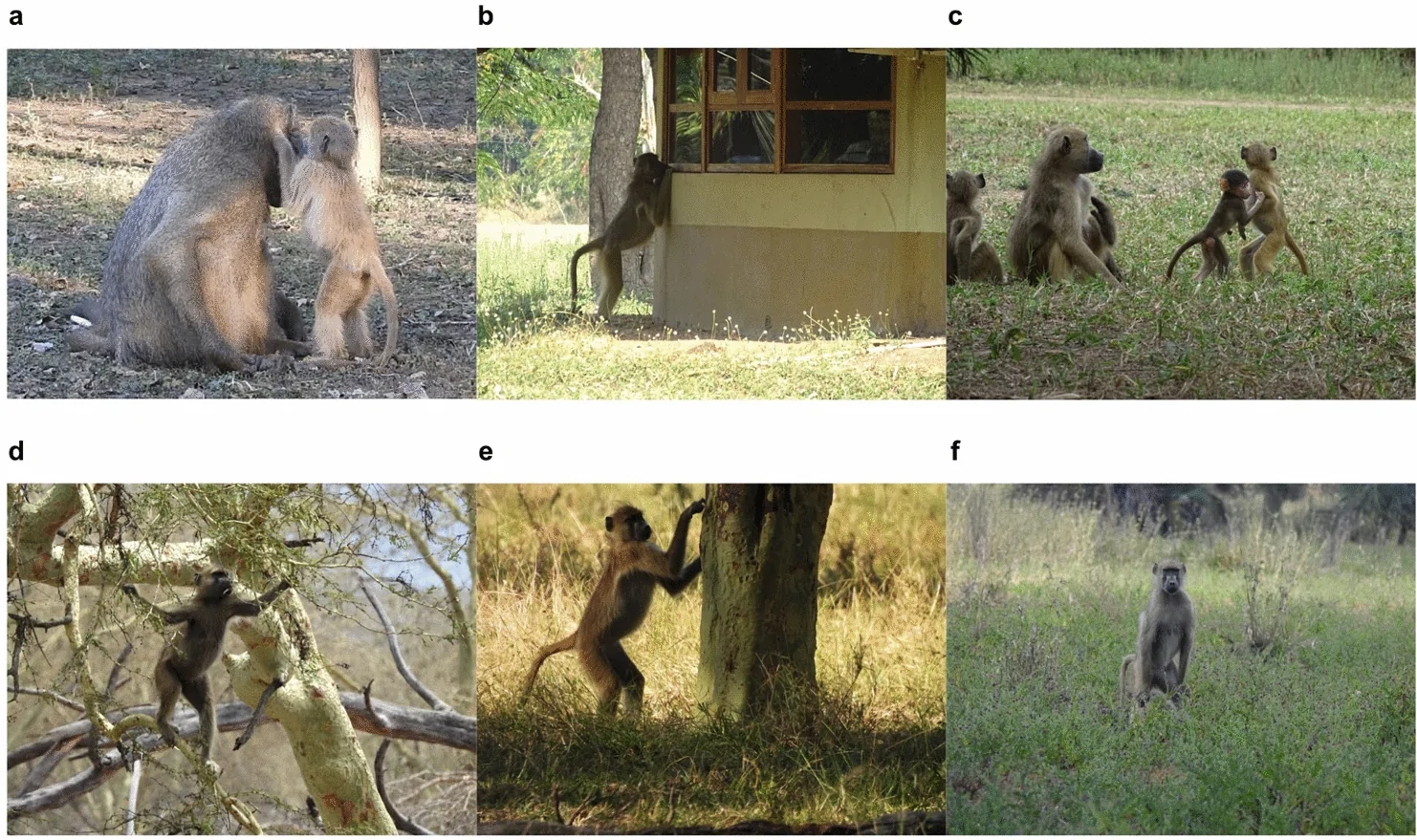
Terrestrial and arboreal bipedalism in chacma baboons. **a** A juvenile grooming an adult in a bipedal posture (photograph by Kerris Chappell-Smith). **b** An adult adopting a bipedal posture while exploring an external stimulus (photograph by Kerris Chappell-Smith). **c** An infant and a juvenile playing in a bipedal stance (photograph by Kerris Chappell-Smith). **d** An adult foraging arboreally in a bipedal position (photograph by Rassina Farassi). **e** A female baboon extracting gum from a tree in a bipedal position (photograph by Susana Carvalho). **f** A female baboon adopting a bipedal vigilance posture (photograph by Rassina Farassi)

Bipedalism was observed in the following contexts: vigilance, allo-grooming, object carrying, foraging, play, exploration, attempted infant snatching, socio-sexual mounting (SSM) and wading (Fig. 1; Table 2). Vigilance contexts were most frequent (63 bouts; 27.3%), followed by play (49 bouts; 21.2%) and exploring (28 bouts; 12.1%). Wading contexts were the least frequent, with one observed bout (0.4%). For 18 bouts (7.8%), the context of bipedalism was unclear to the observer. Bipedal bouts occurring on the ground were observed in all recorded contexts. Meanwhile, five out of seven bouts of arboreal bipedalism occurred in foraging contexts. The context in which the remaining two arboreal bouts occurred could not be identified.

**Table 2:** Contexts of bipedalism

Context	Description of bipedal bout	Bouts (%)
Vigilance	Performed to increase field of vision directed towards an external stimulus	63 (27.3)
Play	Expressed during playful interactions alone or with others, including rough-and-tumble play and acrobatic play	49 (21.2)
Exploring	Investigation of an external (i.e. not part of itself) stimulus using hands. Stimuli may be food-related, but the consumption of food is not realised. Includes ‘exploratory chewing’, where the chewed object does not constitute food	28 (12.10)
Foraging	Performed to obtain food which is subsequently eaten. This includes breastfeeding. It neither includes ‘exploratory chewing’ nor attempted foraging, by which the consumption of food is not realised	20 (8.66)
Unclear	The observer could not identify the context in which bipedalism occurred	18 (7.79)
Infant snatch attempt	Performed in attempt to appropriate an infant from another individual. Does not include successful attempts	16 (7.36)
Carrying	An object/animal is held in one or both hands; if one-handed carrying, the non-carrying hand is not touching any supporting substrate. Includes carrying of conspecifics, including infants appropriated from other individuals	13 (5.63)
Allo-grooming	The focal individual adopts a bipedal posture to groom another	9 (3.90)
Socio-sexual mounting	Bipedal mounting (ventro-dorsal copulation posture) with no reproductive function, i.e., occurring between individuals which belong to the same sex and thus cannot reproduce together, or by pre-reproductive age individuals. This does not include opportunistic mounts by reproductive age males of pre-reproductive age females	9 (3.90)
Other	Included: (1) an infant reaching for its mother, (2) an infant trying to climb onto the back an adult female, (3) an adult male mounting a non-swelling female and (4) a failed attempt by a male to mount a female with visible oestrus swelling (neither of the latter two resulted in copulation)	4 (1.73)
Wading	Walking through water in a bipedal posture	1 (0.43)

### Bipedalism by age-sex class

#### Rates

Model-estimated bipedalism rates ranged from 0.07 to 0.59 bouts per hour of class-specific exposure across age–sex classes (Table S1), with infants/juveniles exhibiting the highest rates and sub-adult females the lowest. Infants/juveniles also had the highest total exposure hours out of all age-sex classes, while sub-adult females had the lowest (Table S2). Bipedalism rates in infants/juveniles were significantly higher than those of adult females, the reference group (rate ratio = 3.52, 95% CI: 1.67–7.41, P = 0.002). After accounting for differences in class-specific exposure and troop, adult males, subadult females, and subadult males showed lower estimated rates relative to adult females, although uncertainty was large and these contrasts were not significant (all P > 0.3; Table 3).

**Table 3:** Effects of age–sex class and troop on bipedalism rates^1^

Predictor	Estimate	95% Confidence interval	P-value
Adult male vs adult female	0.79	0.26–2.40	0.69
Infant/juvenile vs adult female	3.52	1.67–7.41	0.002
Subadult female vs adult female	0.42	0.06–2.90	0.39
Subadult male vs adult female	0.68	0.17–2.66	0.58
Woodland vs Chitengo troop	0.57	0.33–0.99	0.055

^1^Values are rate ratios (events per individual-hour) estimated from a quasi-Poisson generalized linear model with a log link and individual-hour exposure included as an offset. Adult females and the Chitengo troop are the reference categories

#### Contexts

**Fig. 2:**
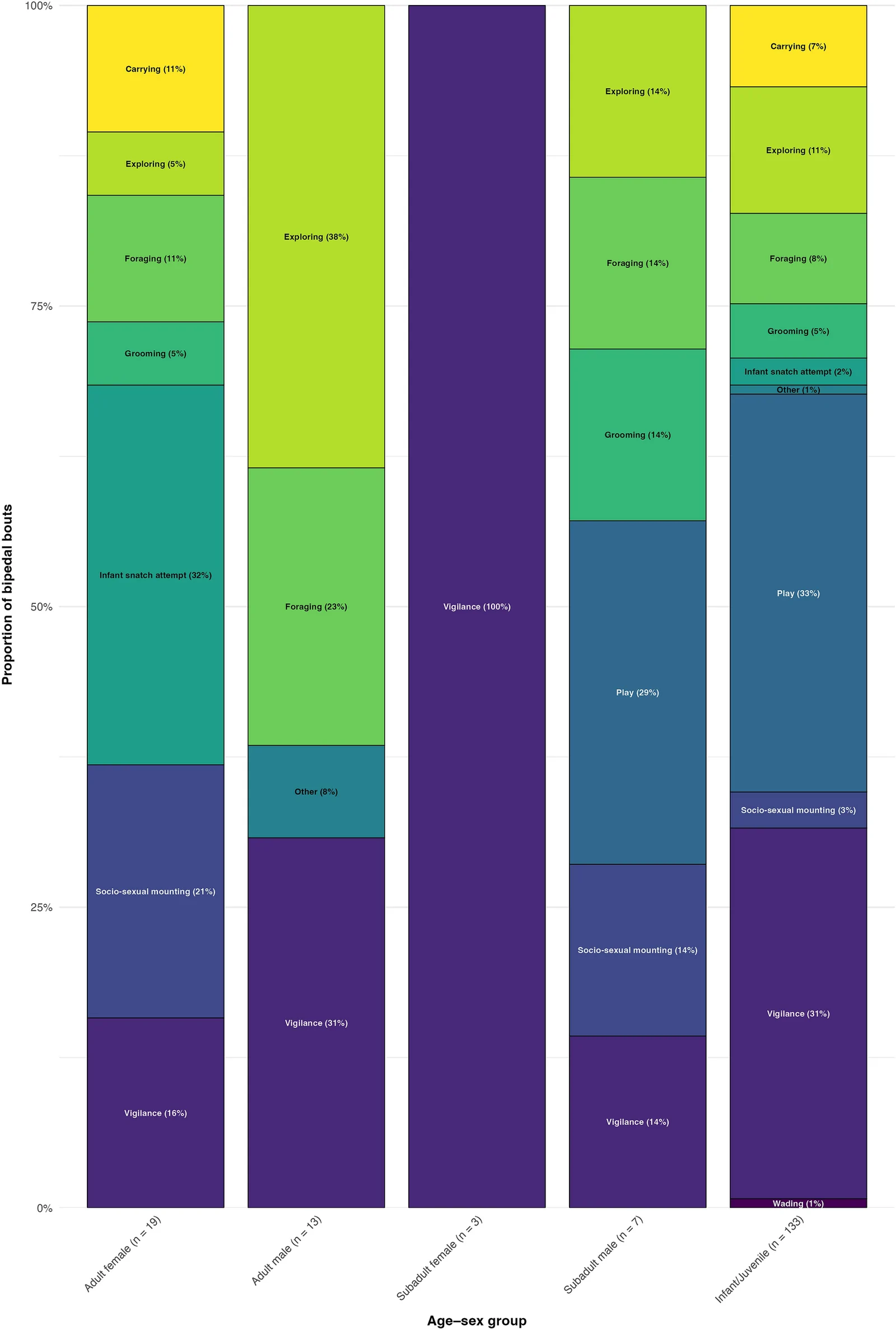
Distribution of bipedalism contexts across age-sex classes. Colors represent each context. The relative contribution of each context to each age-sex class’ bipedal bout count is represented by the proportional height of the segments in each bar (%)

The contexts of bipedalism varied between age–sex classes. Vigilance was the only context reported in all age-sex classes (Fig. 2). Infants/juveniles were the only class for which all contexts were reported and represented the majority of bipedal bouts in several contexts, including play (95.7%), vigilance (78.8%), exploring (66.7%), foraging (62.5%), and grooming (75.0%). Infant snatch attempts were predominantly performed by adult females (66.7%), with the remaining bouts attributed to infants/juveniles. SSM bipedalism was performed by adult females and infants/juveniles (each 44.4%), with a smaller contribution from subadult males (11.1%). The only instance of wading was carried out by an infant/juvenile who was observed wading bipedally in a water pan in Chitengo camp. Subadult females were only seen adopting bipedal postures in a vigilance context.

## Discussion and conclusion

This study provides the first description of bipedal behaviors among chacma baboons (*P. ursinus griseipes*) in the woodland-grassland savanna ecosystem of GNP, Mozambique. Overall, we found that bipedalism occurred in a wide range of contexts, including some which have not, to our knowledge, been proposed as potential drivers of bipedalism in hominins and which are linked to sociality. We also found that the rate and contexts of bipedal behaviors varied between age-sex classes.

Our results support many of the earlier findings from Rose (1976) who reported on the bipedal behaviors of olive baboons near Gilgil, Kenya. Both populations showed mainly postural bipedalism occurring in a wide range of contexts. Also, like Rose (1976), we found that arboreal bipedalism was less frequent than bipedal behavior on the ground (~ 3% versus ~ 92% of bouts) and, when it did occur, it was primarily in a foraging context in the Woodland troop. This may reflect differences in vegetation structure between our two study troops: the Woodland troop inhabited an area with greater tree density than the Chitengo troop, potentially providing more opportunities for arboreal bipedalism. However, bipedalism rates did not differ significantly between the Woodland and Chitengo troops, suggesting that habitat openness does not drive increased bipedal postures or locomotion in woodland-savanna baboons, mirroring findings in woodland-savanna chimpanzees (Drummond-Clarke et al. 2022). Direct testing is needed to determine how tree cover affects baboon bipedalism rates and the rate of terrestrial versus arboreal bipedalism.

In contrast to Rose’s (1976) study which reported feeding as the most common context of bipedalism, vigilance contexts were most frequent in our study. Bipedal vigilance was frequently observed in both the habituated Chitengo troop and the unhabituated Woodland troop, so the difference is likely not due to increased responses to observer presence in Gorongosa compared to Rose’s (1976) habituated troop. Differences between the two sites may be a product of interspecific behavioral diversity, a possibility that demands testing using data from more baboon species across longer observation periods. Alternatively, differences in the prevalence of bipedal vigilance between the two studies may be driven by distinct vegetation densities. The habitat at Gilgil is described as open meadow with scattered trees (Rose 1976), while at least part of the GNP study sites consisted of woodland vegetation. Increased antipredator responses such as vigilance have been reported among baboons in areas with more tree cover compared to open habitats, a response potentially linked to reduced visibility of predators and other dangers (Altmann and Altmann 1970; Cowlishaw 1997; Hammond et al. 2022; Hill and Weingrill 2007; Rasmussen 1983). Therefore, it is possible that Gilgil’s more sparsely wooded environment provided increased visibility and prompted less bipedal vigilance responses than GNP. Future studies should seek to clarify any potential effects of vegetation density on bipedal vigilance.

Along with vigilance, foraging, carrying and wading, which have been proposed by several authors as potential functions of bipedalism in hominins (Amaral 2022; Carvalho et al. 2012; Day 1986; Drummond-Clarke et al. 2022; Etkin 1954; Hardy 1960; Hewes 1961; Isaac 1978; Jolly 1970; Lovejoy 1981; Rose 1976; Washburn 1967; Wrangham 1980; Wrangham et al. 2009), we also observed baboon bipedalism in the following contexts: play, exploring, allo-grooming, SSM and attempted acquisition of an infant. Aside from explorative bipedalism, all of these contexts have also been observed in olive baboons (Owens 1976; Rose 1976),but have rarely been proposed as important functions in hominins.

Many of these additional contexts are social behaviours, and may highlight a potential link between hominin bipedalism and the subsequent evolution of social traits. For instance, play, which was the second most frequent context of bipedalism in our study and the primary context among infants and juveniles (33.1%) and sub-adult males (28.6%), has established functions of both locomotor and social development (Behncke 2015; Fagen 1976; Poirer and Smith 1974). In our study, the form of play appeared to differ depending on vegetation density; in more densely wooded areas, juveniles performed acrobatic chasing play, swinging and jumping through trees, but terrestrial play was predominantly a bipedal ‘wrestling’ form in which playmates gripped each other, typically face-to-face, and grappled. With the contraction of forests in the late Miocene-early Pleistocene (Cerling et al. 2011; Levin 2015; Negash et al. 2024), this terrestrial-bipedal wrestling play may have become the predominant mode in young hominins, not only strengthening bipedal locomotor ability but also shifting the conditions for social bonding between juveniles.

Allo-grooming was also observed as a context of bipedalism among infants/juveniles, sub-adult males and adult females and is a behaviour with well-established social-bonding functions (Dunbar 2010b). While allo-grooming can occur in quadrupedal and seated stances, since it is most frequently a bimanual behaviour (Dunbar 2010b), it may be argued that the adoption of bipedalism allowing for increased efficiency of allo-grooming and thus increased selection on social bonding and related traits. Further research is required to understand whether these additional (social) contexts of bipedalism occur in other populations of baboons and other semi-terrestrial primates.

Our study also suggests a need to consider potential social benefits of functions which have already been proposed by existing hypotheses. In particular, while hypotheses for vigilance bipedalism have traditionally been associated with predator avoidance (Dart 1959), our study as well as Rose (1976) found that most vigilance bouts were directed towards intra-group activities including conflict and group movement. While this may be related to the carnivore-depleted ecosystem of GNP, further study of vigilance bipedalism (including an analysis of stimuli) is required, as there may be a social function of this behaviour that has been largely neglected by current hypotheses.

Finally, our results support observations in wild (Rose 1976) and captive (Druelle & Berillon 2013) olive baboons that the rate and contexts of bipedalism differ between age-sex classes. Infants/juveniles represented the highest proportion of bipedal bouts compared to other age-sex classes and represented the largest proportion of bipedal bouts in most contexts. The only exceptions were SSM and infant snatching, for which adult females represented an equally high (SSM) or higher (infant snatching) proportion of bouts. The high prevalence of bipedalism in early stages of development has been linked to differences in mass distribution between immature and adult baboons (Druelle & Berillon 2013), as well as differences in skeletal maturation and interlimb coordination between immature and adult chimpanzees which facilitate infant/juvenile bipedal postures and locomotion (Doran 1992). That said, between-class differences in rate and context of bipedal behavior may also, to some extent, be a product of disparities in the sampling effort for each age-sex class in our study. Nonetheless, these patterns do introduce the possibility that bipedalism was performed in several contexts in hominin groups, distributed differently according to age-sex class (Druelle & Berillon 2013). Single-function bipedalism hypotheses may thus need reassessing.

### Limitations

This study is based on data collected by a single observer over a short observation period (the Chitengo troop was observed for 39.3 h, and the Woodland troop for 34.3 h; Table 1). There was an uneven sampling effort across baboon age–sex classes, bipedalism in infants and juveniles was not differentiated, and their sex was not identified. The short time frame of this study, combined with the unhabituated state of one of the studied baboon troops, also precluded the identification of studied individuals. Therefore, some degree of non-independence between bipedal observations cannot be excluded and intra-individual variation in bipedal behavior was not assessed. Finally, one of the studied baboon troops was under constant human presence, which may have influenced bipedal behavior. The short time frame of this study also precluded the assessment of seasonal patterns in bipedalism which is a limitation considering that extreme seasonality in rainfall and resource availability is a key characteristic of woodland-grassland savanna environments (Lindshield et al. 2021).

### Conclusion

This study provides the first description of bipedal behavior in chacma baboons. Among its key findings was the observation that bipedalism occurred in a very broad range of contexts, including diverse social contexts which have rarely been proposed as important functions of bipedalism in hominins, but could highlight a potential link between bipedalism and the subsequent evolution of social traits in the human lineage. In addition, the rates and contexts of bipedalism varied between age-sex classes, in some cases to a significant degree.

These findings suggest that bipedalism in chacma in baboons is multi-contextual, demographically structured and socially relevant behavior, and point towards a potential multiple function model of bipedalism in hominins. The existence of multiple, complementary functions of bipedalism would have made the evolution of this trait a stable process: reductions in the frequency/efficiency of any single function would not cease selection for bipedalism altogether, while increases would produce positive feedback, selecting more powerfully for bipedalism. Given the apparently strong selection for bipedalism in the human lineage and the complex suite of traits needed for obligate bipedal locomotion, a multiple-function model for its evolution is logical. A consequence of the differential distribution of bipedal functions across age-sex classes is that the expression of bipedal functions may change over the lifetime of a single individual, as it ages from infancy to reproductive maturity. Similarly, shifts in the age-sex structure of primate groups over time due to social and/or ecological changes may also affect the distribution of bipedal functions. Few theoretical or empirical works propose or test the effect of aging or socio-ecological context on the expression and distribution of bipedal functions, but longer-term studies could bring us closer to understanding if this is relevant to the emergence of bipedalism in the human lineage.

As a limited study with a short observation period, this paper highlights several other areas for future research, including the impact of vegetation density on bipedalism rates and context, as well as studies of intra- and interspecific bipedalism with longer observation periods. Together, the results of this study with those of the recommended future work could provide new perspectives and insights on the emergence and fixation of obligate terrestrial bipedalism, a trait that remains a key defining trait of the human lineage.

## Supplementary Information

Below is the link to the electronic supplementary material.

Supplementary file1 (CSV 22 KB)

Supplementary file2 (CSV 1 KB)

Supplementary file3 (CSV 43 KB)

Supplementary file4 (PDF 90 KB)

Supplementary file5 (PDF 94 KB)

Supplementary file6 (PDF 94 KB)

## Data Availability

All data supporting the results and analyses can be accessed in Supplementary Files S1–S3.
