# Supplementary material for: Bipedality in chacma baboons (Papio ursinus griseipes) in Gorongosa National Park: a multi-contextual, socially-relevant and demographically-structured behavior
Source: Primates. 2026 Jul 17;67(5):567–78. doi: 10.1007/s10329-026-01277-w (PMC13541862; doi:10.1007/s10329-026-01277-w)
Supplement: Supplementary file 1 [file 10329_2026_1277_MOESM4_ESM.pdf]

Article title: Bipedality in chacma baboons (*Papio ursinus griseipes*) in Gorongosa National Park: A multi-contextual, socially-relevant and demographically-structured behavior

Journal name: Primates

Author names: Kerris Chappell-Smith, Dominique Isabel Cavallaro & Susana Carvalho

Corresponding author affiliation: Institute of Human Sciences, University of Oxford, United Kingdom

Corresponding author e-mail address: kerrischappellsmith@gmail.com

Table S1 Model-estimated bipedalism rates by age–sex class

Age-sex class	Rates (events per individual-hour)[1]	95% Confidence interval[2]
Adult female	0.17	0.08–0.34
Adult male	0.13	0.06–0.32
Subadult female	0.07	0.01–0.43
Subadult male	0.11	0.03–0.38
Infant/juvenile	0.59	0.44–0.81

[1]Rates were estimated from a quasi-Poisson generalized linear model with a log link and class-specific exposure (hours) included as an offset. Values represent marginal means averaged over troop.

[2]Confidence intervals are asymptotic 95% intervals back-transformed from the log scale.
