# Supplementary material for: Bipedality in chacma baboons (Papio ursinus griseipes) in Gorongosa National Park: a multi-contextual, socially-relevant and demographically-structured behavior
Source: Primates. 2026 Jul 17;67(5):567–78. doi: 10.1007/s10329-026-01277-w (PMC13541862; doi:10.1007/s10329-026-01277-w)
Supplement: Supplementary file 2 [file 10329_2026_1277_MOESM5_ESM.pdf]

Article title: Bipedality in chacma baboons (*Papio ursinus griseipes*) in Gorongosa National Park: A multi-contextual, socially-relevant and demographically-structured behavior

Journal name: Primates

Author names: Kerris Chappell-Smith, Dominique Isabel Cavallaro & Susana Carvalho

Corresponding author affiliation: Institute of Human Sciences, University of Oxford, United Kingdom

Corresponding author e-mail address: kerrischappellsmith@gmail.com

Table S2 Class-specific exposure hours and number of bipedal observations by age–sex class and troop

**Age-sex class**	**Chitengo troop exposure (h)[1]**	**Chitengo troop total bipedalism observations**	**Woodland troop exposure (h)[1]**	**Woodland troop total bipedalism observations**	**Total exposure (h)[2]**	**Total bipedalism observations**
Adult female	60.0	15	51.7	5	111.7	20
Adult male	52.5	10	36.5	3	89	13
Subadult female	26.7	1	9.0	2	35.7	3
Subadult male	41.5	7	8.51	0	50.01	7
Infant/ juvenile	146.0	113	65.8	31	211.8	144

[1]Estimated by summing session-level age-sex class-specific exposure hours (detailed in Section 2.3) across all sampling sessions within each troop to obtain total exposure hours per age-sex class per troop.

[2] Estimated by summing session-level class specific exposure hours (detailed in Section 2.3) across all sampling sessions and troops to obtain total exposure hours per age-sex class.
