# Supplementary material for: Bipedality in chacma baboons (Papio ursinus griseipes) in Gorongosa National Park: a multi-contextual, socially-relevant and demographically-structured behavior
Source: Primates. 2026 Jul 17;67(5):567–78. doi: 10.1007/s10329-026-01277-w (PMC13541862; doi:10.1007/s10329-026-01277-w)
Supplement: Supplementary file 3 [file 10329_2026_1277_MOESM6_ESM.pdf]

Article title: Bipedality in chacma baboons (*Papio ursinus griseipes*) in Gorongosa National Park: A multi-contextual, socially-relevant and demographically-structured behavior

Journal name: Primates

Author names: Kerris Chappell-Smith, Dominique Isabel Cavallaro & Susana Carvalho

Corresponding author affiliation: Institute of Human Sciences, University of Oxford, United Kingdom

Corresponding author e-mail address: kerrischappellsmith@gmail.com

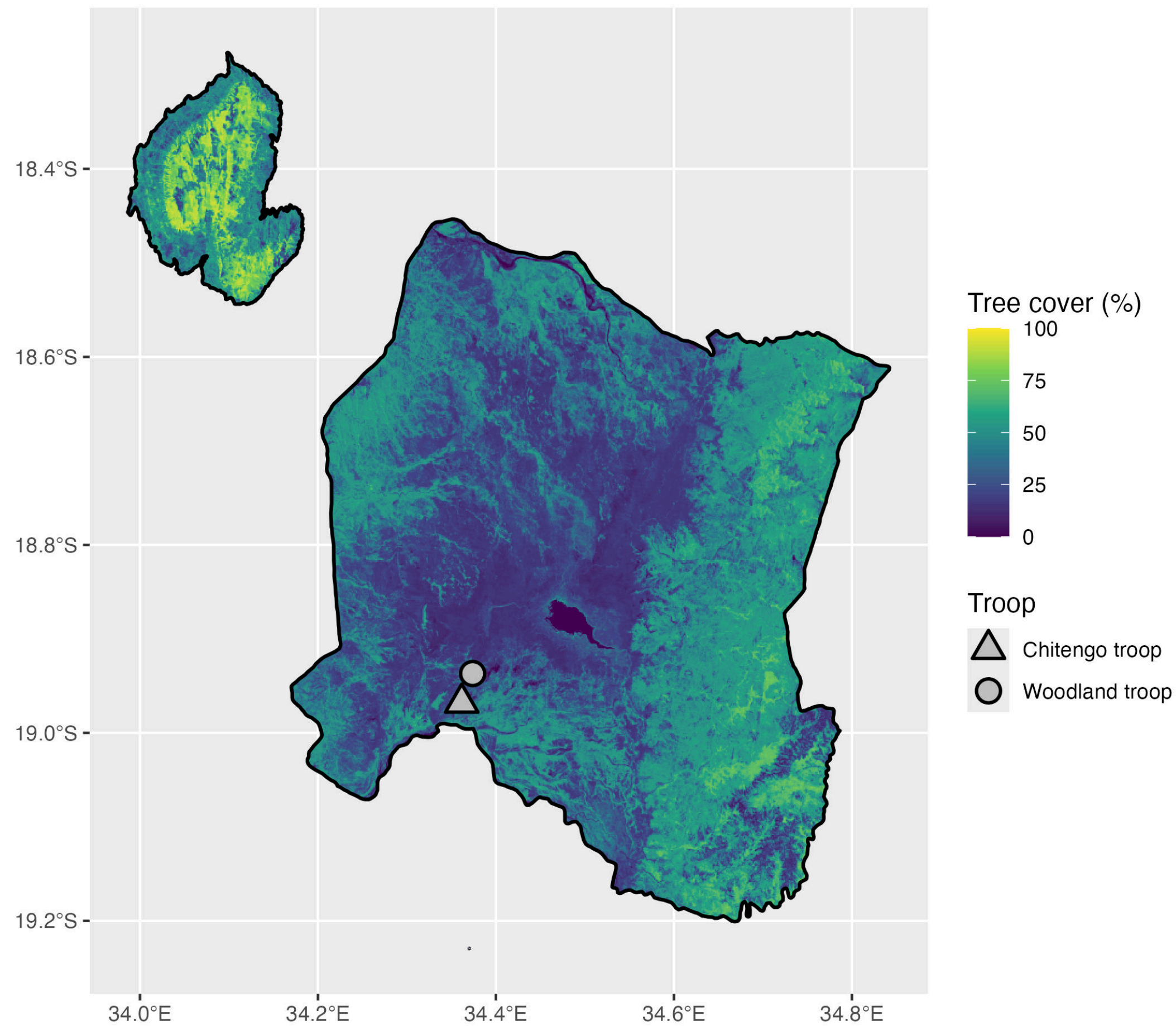

Fig S1 Location of the Woodland and Chitengo baboon troop range in Gorongosa National Park, Mozambique. The triangle marks the location of the Chitengo troop and the circle marks the location of the Woodland troop. The blue to yellow gradient indicates the percentage of tree cover as extracted from the Hansen Global Forest Change dataset, using the treecover2000 layer from version 1.12 (Hansen *et al*. 2013). This layer represents percent tree canopy cover in the year 2000 for vegetation taller than 5 m, at approximately 30 m spatial resolution (Hansen *et al*. 2013).
